# Association between Heat Vulnerability Index and Stroke Severity

**DOI:** 10.3390/ijerph21081099

**Published:** 2024-08-20

**Authors:** Jason J. Wang, Jeffrey M. Katz, Maria X. Sanmartin, Liron D. Sinvani, Jason J. Naidich, Elizabeth Y. Rula, Pina C. Sanelli

**Affiliations:** 1 Northwell Health, New Hyde Park, NY 11040, USA; jkatz2@northwell.edu (J.M.K.); msanmartin@northwell.edu (M.X.S.); ldanay@northwell.edu (L.D.S.); psanelli@northwell.edu (P.C.S.); 2 Institute of Health System Science, The Feinstein Institutes for Medical Research, Manhasset, NY 11030, USA; 3 Departments of Radiology, Donald and Barbara Zucker School of Medicine at Hofstra/Northwell, Hempstead, NY 11549, USA; 4 Departments of Neurology, Donald and Barbara Zucker School of Medicine at Hofstra/Northwell, Hempstead, NY 11549, USA; 5 Departments of Medicine, Donald and Barbara Zucker School of Medicine at Hofstra/Northwell, Hempstead, NY 11549, USA; 6 Harvey L. Neiman Health Policy Institute, Reston, VA 20191, USA; erula@neimanhpi.org

**Keywords:** heat vulnerability index, stroke severity, acute ischemic stroke

## Abstract

Background: Socioeconomically disadvantaged neighborhoods are particularly vulnerable to heat-related illnesses. We aim to investigate the association between the heat vulnerability index (HVI), an established neighborhood-level metric of heat-related mortality risk, and acute ischemic stroke (AIS) severity. Methods: We conducted a retrospective analysis of consecutive AIS admissions to a comprehensive stroke center between 2012 and 2021. Stroke severity was defined upon admission based on the National Institutes of Health Stroke Scale (NIHSS). Demographic, socioeconomic, and clinical characteristics were extracted from electronic health records. HVI status was assigned using residential ZIP codes. Multivariable logistic regression analyses were performed. Results: Of 3429 AIS admissions, 1123 (32.8%) were from high-HVI (scores 4–5) neighborhoods and 868 (25.3%) had severe stroke (NIHSS score ≥ 10). In the multivariable regression model with stepwise selection, a high HVI was independently associated with severe stroke (adjusted odds ratio: 1.40 [95% confidence interval 1.16–1.69]). Conclusions: The association between a high HVI and severe stroke underscores the importance of targeting policy interventions to mitigate heat-related illness in socioeconomically disadvantaged neighborhoods.

## 1. Introduction

Acute ischemic stroke (AIS) is a leading cause of disability and death in the United States [1,2]. AIS disproportionately affects socioeconomically disadvantaged groups and results in worse clinical outcomes [3,4]. In particular, low-income and racial minority (i.e., Black and Hispanic) groups have higher rates of AIS [5] and more severe strokes [6], and are associated with higher rates of mortality and dependent disabilities [7]. Other disparities in stroke care have also been described based on geographic factors. AIS patients residing in rural, compared to urban, settings have been found to have lower utilization of stroke imaging and acute treatment [8]. Furthermore, there was significantly lower imaging utilization in the southern region of the United States, suggesting regional variability in practice patterns and resource utilization [8]. Thus, studying the geographic and environmental factors leading to inequities in stroke outcomes will provide further insights for addressing disparities in stroke care.

Environmental heat exposure is an AIS risk factor, with higher temperatures linked to increased AIS incidence and mortality [9]. Previous studies primarily focused on biological (e.g., hypertension) and intrapersonal (e.g., lifestyle) determinants [4,8]. Socioeconomically disadvantaged neighborhoods are particularly vulnerable to heat-related illnesses due to limited availability of the resources needed to mitigate the effects of heat, yet no studies have evaluated the combined impact of heat and socioeconomic disparity on AIS severity [10].

The heat vulnerability index (HVI) is an established metric incorporating factors associated with adverse health effects during extreme heat and is used to identify neighborhoods with an elevated risk of heat-related deaths [11]. The New York City (NYC) Department of Health developed the HVI using a statistical model incorporating environmental (i.e., surface temperature and green space) and social (i.e., poverty and race) factors, with a scoring system from 1 (low risk) to 5 (high risk) [12], while similar indexes were developed for other regions in the nation [13]. Vulnerability to heat geographically varied in NYC neighborhoods [12]. The socioeconomically disadvantaged neighborhoods experienced the highest vulnerability, with increased heat stress emergency events [13]. Since susceptibility to heat stress depends on interrelated environmental and social factors, the HVI is a valuable metric to study neighborhood-level disparities.

Examining individual factors alone may not provide a complete understanding of heat-related disparities. Neighborhoods with resources to combat heat, such as air-conditioned spaces, experience lower vulnerability. By considering both environmental and social determinants using the HVI, nuanced insights into heat-related disparities can be gained to inform policymaking efforts to reduce stroke disparities. Exploring neighborhood factors using a novel modifiable stroke risk factor for AIS is an innovative approach to address environmental and social determinants of stroke disparities.

This study aims to explore the association between the HVI and stroke severity, as measured by the National Institutes of Health Stroke Scale (NIHSS) at admission, leveraging a comprehensive database from a diverse socioeconomic region in the NYC metropolitan area. We hypothesize that patients from high-HVI neighborhoods have worse stroke severity at presentation compared to patients from low-HVI neighborhoods. Our research builds upon previous work on stroke disparities [4,8,14], aiming to identify and address geographic-level inequities related to neighborhood characteristics. By extending our investigation to explore novel avenues in stroke research, we strive to contribute to reducing disparities in stroke care delivery and outcomes.

## 2. Materials and Methods

### 2.1. Study Design and Cohort

The study cohort was extracted from the Stroke Health Outcomes (SHOUT) database, representing a retrospective, consecutive series of AIS admissions to a comprehensive stroke center (CSC) serving NYC between 1 January 2012 and 31 December 2021. Our CSC functions as the hub of a large, integrated, urban–suburban stroke network, composed of 17 stroke centers. Patient information was imported into the SHOUT database from the American Heart Association Get-With-The-Guidelines-Stroke database based on discharge diagnosis codes for ischemic stroke (International Classification of Disease 9/10: 433.x1, 434.x1, I63.xx). The socioeconomic information was self-reported by patients or caregivers at admission.

The inclusion criteria were (1) adult patients (≥18 years old) with (2) discharge codes for ischemic stroke (International Classification of Disease 9/10 433.x1, 434.x1, I63.xx). The exclusion criteria were (1) emergency visits that did not yield hospitalization and (2) a residential zip code missing or outside of NYC. We obtained Institutional Review Board approval with a waiver of consent given the retrospective nature of this study.

### 2.2. Data Collection

Individual-level variables were collected with the following categorization: (a) demographic: age (18–49, 50–79, and 80+ years), gender (female, male), race (Asian, Black, white, other/unknown) based on self-identification and categorized according to the National Institutes of Health policy [15], insurance type (Medicare, Medicaid, private, uninsured), and median household income (<USD 80,000, USD 80,000+) based on patient zip codes reported by the United States Census Bureau [16]; (b) arrival method (ambulance, private transportation, transfer from primary stroke center, in-hospital stroke, unknown); (c) clinical: NIHSS score, last known well to arrival (LKWA) time (<6 h, 6–24 h, >24 h, in-hospital stroke, unknown), and medical comorbidities (atrial fibrillation, coronary artery disease, carotid stenosis, depression, diabetes mellitus, drug/alcohol abuse, dyslipidemia, family history of stroke, hypertension, obesity, peripheral vascular disease, previous stroke, renal insufficiency, sleep apnea, tobacco smoking, transient ischemic attack, and none).

We acquired HVI data from the NYC Department of Health. Using residence zip codes, we merged the study cohort with NYC-HVI data. The NYC-HVI scores ranged from 1 (lowest risk) to 5 (highest risk). We dichotomized the HVI groups into low-HVI (scores 1–3) and high-HVI (scores 4–5).

### 2.3. Statistical Analysis

Descriptive frequency statistics and bivariate analyses were conducted using Chi-square tests to assess significant differences in the demographic and clinical characteristics between HVI groups. For continuous variables (age, NIHSS, LKWA), the mean, standard deviation (SD), median, and interquartile range (IQR) were also calculated. *p*-values were estimated for categorical groups using the Chi-square test and for the mean values using *t*-tests.

Separate logistic regression analyses were conducted to evaluate the associations between a high HVI and severe stroke, with the dependent variable being the NIHSS score upon admission. The primary outcome was severe stroke, defined as an NIHSS score ≥ 10. Analyses were performed utilizing other NIHSS cut-offs to define “severe” stroke (NIHSS score ≥ 6 and ≥16) [4,8,17]. We also performed separate analyses to account for patients with missing admission NIHSS scores (15.5%) by either including them with the low NIHSS category [14] or excluding them from the analyses. Independent variables were discharge year (2012–2021), demographics (excluding race since it is a component of the HVI), arrival method, LKWA, and comorbidities. Patients with missing data for LKWA (27.1%) were grouped with the >24 h category for out-of-hospital stroke onset [4,8]. To avoid multi-collinearity, we performed multivariable regression analyses using stepwise selection while maintaining a high HVI in the models.

Odds ratios (ORs), 95% confidence intervals (CIs), and *p*-values were estimated. *p*-values < 0.05 were considered statistically significant. SAS v9.4 software (SAS, Cary, NC, USA) was used for all statistical analyses.

## 3. Results

The demographic and clinical characteristics of the study cohort are presented in Table 1. Of 7540 AIS episodes from 2012 to 2021 in the SHOUT database, 3429 (45.5%) were from the NYC area. The number of annual AIS episodes ranged from 170 visits in 2012 to 490 visits in 2021. Amongst the 3429 AIS visits, 32.8% were from high-HVI (4–5) neighborhoods, 31.8% were aged 80+, 49% were female, 23.1% were Black, 13.8% had an LKWA time of 24+ h, and 25.3% had an NIHSS score of 10+.

As shown in Table 1, we also performed statistical comparisons between low- and high-HVI categories. Bivariate analyses show that a high HVI was significantly associated with higher stroke severity (NIHSS score 10+: 28.6% vs. 23.7%, *p* = 0.004). A high HVI was also significantly associated with younger age (mean [years] 66.4 vs. 72.8, *p* < 0.0001), Black race (59.1% vs. 5.6%, *p* < 0.0001), Medicaid insurance (23.1% vs. 19.1%, *p* = 0.0072), and a lower MHI (MHI USD 80k+: 32.7% vs. 42.1%, *p* < 0.0001). In terms of stroke risk factors, the high-HVI group also had statistically higher rates of diabetes mellitus (43.8% vs. 31.6%, *p* < 0.0001), hypertension (77.7% vs. 69.6%, *p* < 0.0001), and obesity (44.7% vs. 35.6%, *p* < 0.0001).

Using various NIHSS cut-off points and excluding the missing data, we found that a high HVI (4–5) was a statistically significant risk factor in all logistic regression models (Table 2), with an NIHSS score ≥ 10 representing the most significant one (OR = 1.40; 95% CI = [1.16, 1.69]). We also ran a regression model with the missing NIHSS data included in the reference group and tested this using a score of 5 as a high HVI. All of the regression models showed a significant impact of a high HVI on stroke severity, with similar odds ratios (ranging from 1.26 to 1.40).

As indicated in Table 3, factors such as older age, female gender, transferred patients, arrival via emergency medical services (EMSs), an LKWA time of <24 h, atrial fibrillation, and drug/alcohol use were associated with higher stroke severity (NIHSS score of 10+). Conversely, carotid stenosis, smoking status, and transient ischemic attack were associated with lower stroke severity.

## 4. Discussion

To our knowledge, this is the first study to report an association between the HVI (metric of combined environmental and socioeconomic factors) and stroke severity. The association between a high HVI and severe AIS was significant regardless of the NIHSS threshold studied (≥6, ≥10, or ≥16), with similar odds ratios. Although further investigation is warranted, our findings potentially represent a novel, modifiable, neighborhood-level stroke risk factor. Our study also uniquely describes the demographic and clinical characteristics of AIS patients from high-HVI neighborhoods.

Our study pioneers the evaluation of the HVI as a potentially new metric for studying stroke disparities by delving into local geographic inequities and exploring interrelated environmental and social factors contributing to stroke severity.

Despite its strengths, our study has certain limitations. It is based on data from a single comprehensive stroke center (CSC) serving a densely populated but geographically confined urban area in NYC. Extending this study to suburban or rural areas may yield different results, necessitating further research. Also, since we do not have the healthcare access information at the patient level, which may be a confounder of the analysis, it is still a limitation. Additionally, the retrospective nature of our study led to missing data, particularly concerning baseline NIHSS scores and LKWA time. Although we implemented methods to mitigate this weakness, such as exploring the analysis with or without the missing data and obtaining similar results, it remains a limitation. Further research with more complete data is needed to ensure the robustness of the conclusions.

Our investigation expands the scope of stroke research by delving into geographic inequities and exploring the interrelated environmental and social factors contributing to stroke severity. Unlike previous studies concentrating on system-level inequities in stroke care access and delivery, we pivot toward studying the neighborhood environment. This shift is paramount, considering the escalating concern over rising temperatures and the existing disparities in stroke and heat-related deaths. By focusing on neighborhoods vulnerable to the health effects of heat, our study aims to provide crucial insights for addressing stroke disparities.

## 5. Conclusions

We identified a novel neighborhood-level marker to evaluate stroke risk. Focusing stroke interventions on modifiable factors contributing to a high HVI, such as heat mitigation, may be an effective public health strategy to reduce stroke burden in socioeconomically disadvantaged neighborhoods. Further research is needed to confirm this association and evaluate whether the HVI also impacts stroke outcomes.

## Figures and Tables

**Table 1:** Demographic and clinical characteristics of study cohort.

Characteristics	Categories		n	%	Low HVI 1–3	High HVI 4–5	*p*-Value
					Mean %	Mean %	
Total			3429	100	67.2	32.8	
Discharge Year	2012		170	5.0	5.8	3.3	<0.0001
	2013		246	7.2	7.9	5.8	
	2014		268	7.8	8.6	6.1	
	2015		326	9.5	10.2	8.2	
	2016		349	10.2	10.8	9.0	
	2017		367	10.7	10.8	10.5	
	2018		387	11.3	10.3	13.3	
	2019		418	12.2	11.4	13.5	
	2020		408	11.9	10.8	14.2	
	2021		490	14.3	13.4	16.1	
Age	18–49 Yrs		311	9.1	7.4	12.4	<0.0001
	50–79 Yrs		2029	59.2	54.6	68.6	
	80+ Yrs		1089	31.8	38.0	19.0	
	Mean		3429	70.7	72.82	66.38	<0.0001
Gender	Female		1681	49.0	48.0	51.0	0.1019
	Male		1748	51.0	52.0	49.0	
Race	Asian		456	13.3	14.8	10.2	<0.0001
	Black		792	23.1	5.6	59.1	
	White		1573	45.9	62.8	11.1	
	Other/Unknown		608	17.7	16.8	19.6	
Insurance	Medicare	No	1559	45.5	40.8	55.1	<0.0001
		Yes	1870	54.5	59.2	44.9	
	Medicaid	No	2729	79.6	80.9	76.9	0.0072
		Yes	700	20.4	19.1	23.1	
	Private	No	1222	35.6	35.4	36.2	0.6598
		Yes	2207	64.4	64.6	63.8	
	Uninsured	No	3328	97.1	97.4	96.3	0.0548
		Yes	101	3.0	2.6	3.7	
Arrival Method	EMSs		959	28.0	31.4	20.9	<0.0001
	Private Transportation		1121	32.7	33.5	31.0	
	Transfer		895	26.1	23.6	31.3	
	In-Hospital Stroke		275	8.0	6.9	10.3	
	Unknown		179	5.2	4.6	6.5	
LKW to Arrival	In-Hospital Stroke		275	8.0	6.9	10.3	0.0023
	0–6 h		874	25.5	26.3	23.9	
	6–24 h		877	25.6	26.6	23.5	
	≥24 h		474	13.8	13.7	14.0	
	Unknown		929	27.1	26.5	28.3	
	Mean		2225	19.3	18.57	20.96	0.1116
Comorbidities	Atrial Fibrillation		579	16.9	18.8	12.9	<0.0001
	CAD		599	17.5	17.9	16.7	0.3794
	Carotid Stenosis		72	2.1	2.2	1.9	0.5126
	Depression		117	3.4	4.0	2.2	0.0076
	Diabetes		1220	35.6	31.6	43.8	<0.0001
	Drug/Alcohol		95	2.8	2.4	3.5	0.0803
	Dyslipidemia		1398	40.8	41.4	39.5	0.3052
	Family Stroke History		60	1.8	2.0	1.3	0.1969
	Hypertension		2478	72.3	69.6	77.7	<0.0001
	Obese/Overweight		1322	38.6	35.6	44.7	<0.0001
	PVD		47	1.4	1.3	1.6	0.4145
	Previous Stroke		656	19.1	18.1	21.2	0.0321
	Renal Insufficiency		220	6.4	5.3	8.6	0.0002
	Sleep Apnea		46	1.3	1.3	1.4	0.7674
	Smoker		308	9.0	8.7	9.6	0.3642
	TIA		137	4.0	4.0	4.0	0.9804
	None		222	6.5	7.7	3.9	<0.0001
NIHSS Score	Unknown		531	15.5	15.0	16.5	0.0091
	0–5		1690	49.3	51.1	45.5	
	6–9		340	9.92	10.2	9.4	
	10–15		345	10.1	9.5	11.1	
	16–42		523	15.3	14.2	17.5	
	Mean		2898	7.4	7.05	8.14	0.0006
HVI	1		914	26.7			
	2		806	23.5			
	3		586	17.1			
	4		773	22.5			
	5		350	10.2			

HVI, heat vulnerability index; EMSs, emergency medical services; LKWA, last known well to arrival; CAD, coronary artery disease; PVD, peripheral vascular disease; TIA, transient ischemic attack; NIHSS, National Institutes of Stroke Scale. *p*-values for categorical groups were calculated with the Chi-square test; *p*-values for the means were calculated with *t*-tests.

**Table 2:** Logistic regression analyses of high-HVI impact on stroke severity using various NIHSS thresholds. Separate analyses were performed to handle patients with missing NIHSS data by either including these patients in low NIHSS group (**A**) or excluding these patients from analysis (**B**).

	High HVI (Scores 4–5)			
Stroke Severity	OR	95% CI		*p*-Value
A. Missing NIHSS Data Included in Reference Low NIHSS Group (N = 3429)				
NIHSS score ≥ 6	1.26	1.07	1.49	0.0067
NIHSS score ≥ 10	1.36	1.14	1.63	0.0009
NIHSS score ≥ 16	1.33	1.03	1.73	0.0300
**B. Missing NIHSS Data Excluded (N = 2898)**				
NIHSS score ≥ 6	1.29	1.08	1.54	0.0045
NIHSS score ≥ 10	1.40	1.16	1.69	0.0005
NIHSS score ≥ 16	1.34	1.03	1.74	0.0302

HVI, heat vulnerability index; NIHSS, National Institutes of Health Stroke Scale; OR, odds ratio; CI, confidence interval.

**Table 3:** Logistic regression analysis with stepwise selection of high-HVI impact on stroke severity (NIHSS score ≥ 10).

Factors	OR	95% CI		*p*-Value
High HVI (scores 4–5)	1.40	1.16	1.69	0.0005
Year 2012	Reference			
Year 2013	0.45	0.31	0.66	<0.0001
Year 2020	1.46	1.12	1.90	0.005
Age ≥ 80	1.34	1.09	1.64	0.0046
Female	1.34	1.12	1.61	0.0012
Transferred	4.60	3.73	5.68	<0.0001
Arrived Using EMSs	1.79	1.44	2.23	<0.0001
Medicaid	1.37	1.11	1.70	0.0037
LWKA time ≥ 24 h	0.44	0.37	0.54	<0.0001
Atrial Fibrillation	1.86	1.49	2.33	<0.0001
CAD	1.31	1.04	1.65	0.0222
Carotid Stenosis	0.36	0.16	0.85	0.0186
Drug/Alcohol	2.09	1.24	3.52	0.0055
Smoker	0.58	0.41	0.82	0.0017
TIA	0.52	0.31	0.88	0.0142

HVI, heat vulnerability index; NIHSS, National Institutes of Health Stroke Scale; OR, odds ratio; CI, confidence interval; EMSs, emergency medical services; LKWA, last known well to arrival; CAD, coronary artery disease; PVD, peripheral vascular disease; TIA, transient ischemic attack. Independent variables not selected by stepwise selection were eliminated from the table. We also adjusted the models by year (using Year 2012 as the reference) to reflect possible time-related impacts (such as COVID-19 in 2020) on the stroke severity of admitted AIS patients.

## Data Availability

The data presented in this study are available on request from the corresponding author due to ongoing studies.

## Abbreviations

HVI = heat vulnerability index; AIS = acute ischemic stroke; MHI = median household income; LKWA = last known well to arrival; NIHSS = National Institutes of Health Stroke Scale; EMSs = emergency medical services; CAD = coronary artery disease; PVD = peripheral vascular disease; TIA = transient ischemic attack.
